# Artificial Intelligence in the Detection of Barrett's Esophagus: A Systematic Review

**DOI:** 10.7759/cureus.47755

**Published:** 2023-10-26

**Authors:** Akash Patel, Gagandeep Singh Arora, Mona Roknsharifi, Parneet Kaur, Hamna Javed

**Affiliations:** 1 Internal Medicine, Eisenhower Health, Rancho Mirage, USA; 2 Hepatobiliary Pancreatic Surgery and Liver Transplant, BLK-Max Super Speciality Hospital, New Delhi, IND; 3 Internal Medicine, University of California, Riverside, San Bernardino, USA; 4 Emergency, Civil Hospital, Mukerian, IND; 5 Internal Medicine, Suburban Community Hospital, Philadelphia, USA; 6 Internal Medicine, Saint Agnes Medical Center, Fresno, USA

**Keywords:** barrett's esophagus, clinical gastroenterology, artificial intelligence in healthcare, barrett's esophagus (be), general internal medicine, internal medicine, artificial intelligence in gastroenterology, ai and machine learning, artificial intelligence in medicine, deep learning artificial intelligence

## Abstract

Barrett's esophagus (BE) remains a significant precursor to esophageal adenocarcinoma, requiring accurate and efficient diagnosis and management. The increasing application of machine learning (ML) technologies presents a transformative opportunity for diagnosing and treating BE. This systematic review evaluates the effectiveness and accuracy of machine learning technologies in BE diagnosis and management by conducting a comprehensive search across PubMed, Scopus, and Web of Science databases up to the year 2023. The studies were organized into five categories: computer-aided systems, natural language processing and text-based systems, deep learning on histology and biopsy images, real-time and video analysis, and miscellaneous studies. Results indicate high sensitivity and specificity across machine learning applications. Specifically, computer-aided systems showed sensitivities ranging from 84% to 100% and specificities from 64% to 90.7%. Natural language processing and text-based systems achieved an accuracy as high as 98.7%. Deep learning techniques applied to histology and biopsy images displayed sensitivities up to greater than 90% and a specificity of 100%. Furthermore, real-time and video analysis technologies demonstrated high performance with assessment speeds of up to 48 frames per second (fps) and a mean average precision of 75.3%. Overall, the reviewed literature underscores the growing capability and efficiency of machine learning technologies in diagnosing and managing Barrett's esophagus, often outperforming traditional diagnostic methods. These findings highlight the promising future role of machine learning in enhancing clinical practice and improving patient care for Barrett's esophagus.

## Introduction and background

Barrett's esophagus (BE) represents a significant public health concern due to its association with esophageal adenocarcinoma, a form of cancer that has been increasing in incidence in Western countries over the past few decades [1]. Originating in the columnar-lined lower esophagus, BE is the consequence of long-standing gastroesophageal reflux disease (GERD). In this precancerous condition, the normal stratified squamous epithelium is replaced by columnar epithelium with intestinal metaplasia. This altered cellular structure puts patients at a substantially higher risk for developing dysplasia and eventually esophageal adenocarcinoma [2]. Despite advances in endoscopic techniques and pharmacological interventions, the diagnosis and management of BE continue to present clinical challenges. In particular, the traditional methods often depend on the operator's expertise, leading to issues related to sensitivity and specificity. Additionally, there are significant costs associated with long-term endoscopic surveillance and management [3].

In light of these challenges, emerging technologies such as artificial intelligence (AI) may offer a potentially transformative approach to improving BE care. AI technologies, including but not limited to machine learning, deep learning, and natural language processing, can process large volumes of data and recognize complex patterns, thereby aiding clinicians in diagnosis and treatment planning [4]. Preliminary research has shown promising results, suggesting that AI could enhance the sensitivity and specificity of BE detection and even help in staging dysplasia or neoplasia in identified cases [5].

Given the evolving landscape of AI applications in healthcare, there is a need for a systematic and comprehensive review to evaluate its role specifically in the context of BE. The potential for AI to revolutionize current diagnostic and therapeutic paradigms makes it imperative to understand its capabilities and limitations thoroughly. While some studies have already explored these aspects, there is a lack of consensus regarding the effectiveness and applicability of AI technologies in routine clinical practice for BE.

This systematic review aspires to compile and assess current findings, future trends, and untapped potentials in applying AI to endoscopic procedures for BE. It will delve into the possibilities for AI to alleviate the often problematic human-induced variability in diagnostic imaging and endoscopy. With the ongoing advancements in AI algorithms, including convolutional neural network (CNN), the technology is setting the stage for radical improvements in BE diagnosis and, subsequently, patient care.

## Review

Methods

Study Design

This systematic review has been meticulously structured to conform to the Preferred Reporting Items for Systematic Reviews and Meta-Analyses (PRISMA) guidelines. The principal objective of this review is to assess the sensitivity and specificity of various artificial intelligence (AI) technologies in the diagnosis and management of Barrett's esophagus (BE).

Eligibility Criteria

The inclusion criteria for this review encompass peer-reviewed original research articles published in English, which focus explicitly on the role of AI technologies such as machine learning, deep learning, and natural language processing in BE. We have restricted our scope to human subject studies that offer clear outcomes pertaining to the sensitivity and specificity of AI applications in diagnosing or managing BE. Moreover, only those studies that provide explicit reporting regarding sensitivity, specificity, accuracy, and other diagnostic parameters have been included. Exclusion criteria for this study are non-English articles, studies conducted on non-human subjects, articles for which the full text was unavailable, and editorials, letters, conference papers, and reviews. Additionally, studies that did not focus explicitly on BE were excluded.

Information Sources and Search Strategy

To ensure a comprehensive literature survey, multiple databases such as PubMed, MEDLINE, EMBASE, and Google Scholar were queried. Search terms included "machine learning," "artificial intelligence," "deep learning," "Barrett's esophagus," and "Barrett's neoplasia," either used in isolation or combined. The search period was restricted to articles published up until October 2023. Additional articles were identified through a manual search involving citation tracking of the studies and reviews initially found.

Study Selection

**Figure 1:**
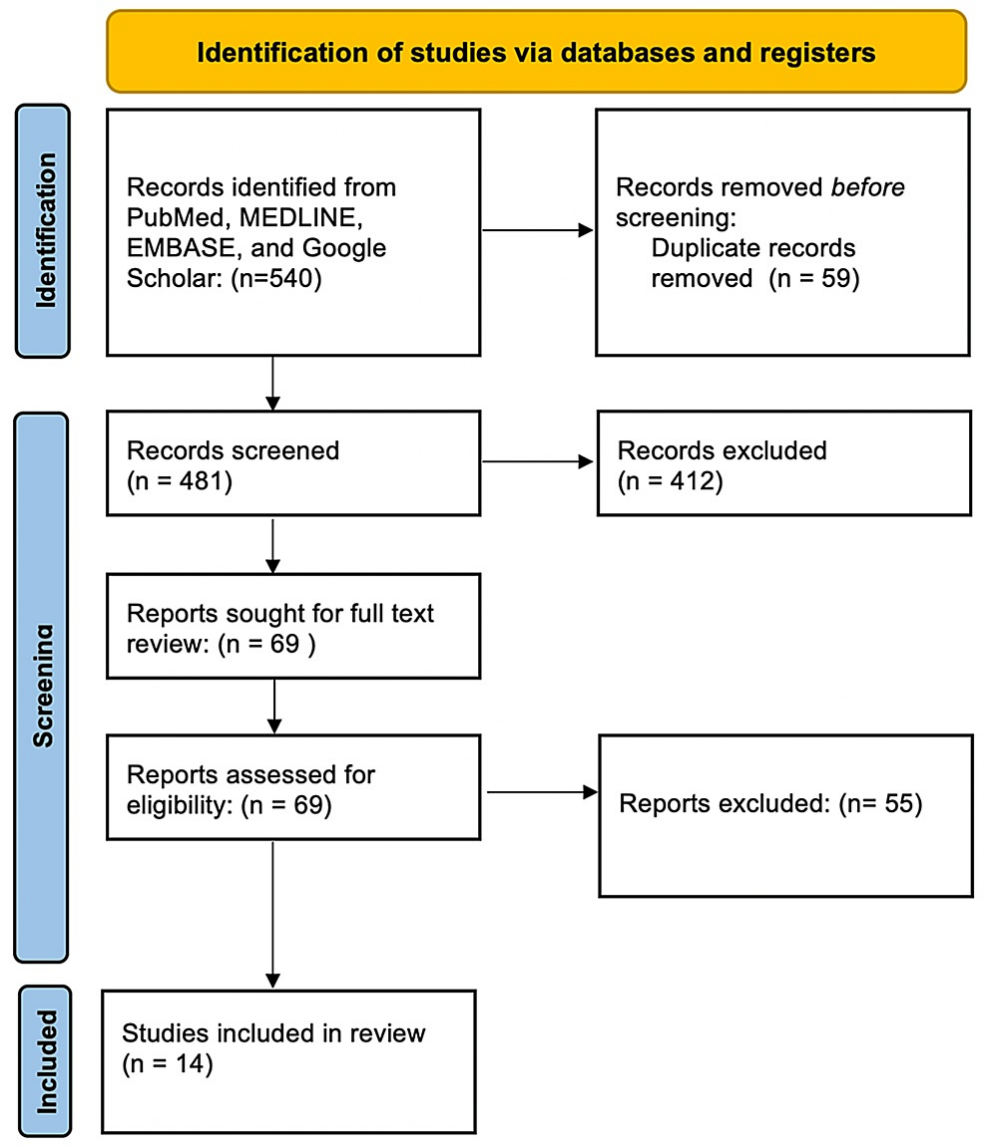
PRISMA structure for the included studies PRISMA: Preferred Reporting Items for Systematic Reviews and Meta-Analyses

The initial literature search yielded a total of 540 articles. After removing 59 duplicates, two independent reviewers screened the titles and abstracts based on the predetermined eligibility criteria. A total of 69 articles were selected for a detailed full-text review. After applying the inclusion and exclusion criteria, a final total of 14 articles were selected for this systematic review. Figure 1 shows our PRISMA structure for study selection.

Data Extraction

Two independent reviewers employed a standardized data extraction form to collect pertinent information from the selected articles. Categories for data extraction included author information, publication year, study objective, methodologies used, sample size, data types, primary outcomes, sensitivity, specificity, other relevant metrics, and study conclusions. Any disagreements between reviewers were settled through discussion, and if necessary, a third reviewer was consulted.

Quality Assessment

Given the nascent and rapidly evolving nature of AI in healthcare, traditional tools for quality assessment are not fully equipped to assess the quality of AI studies. Hence, while the quality of the included studies is acknowledged as a crucial factor, a formal quality assessment was not conducted for this review.

Data Synthesis

Since this is a systematic review that aimed to provide a comprehensive understanding of the existing literature rather than a meta-analysis, data were not combined. The primary aim of data synthesis was to qualitatively summarize the sensitivity and specificity of AI technologies in detecting BE. These findings are narratively synthesized and presented in the Results and Discussion sections of this review.

Results

Study Characteristics

Our systematic review encompasses 14 studies, summarized in Table 1. The studies are categorized based on the type of AI systems employed. These categories include computer-aided detection (CADe) systems, deep learning on histology and biopsy images, real-time and video analysis, natural language processing (NLP) and text-based systems, and miscellaneous studies. Data types across the studies varied, ranging from histology slides and endoscopic images to pathology reports and videos. The data size in these studies also varied considerably, reflecting different scales of validation.

**Table 1:** Overview of studies on AI applications in Barrett's esophagus AI: artificial intelligence, CADe: computer-aided detection, WLE: white light endoscopy, pCLE: probe-based confocal laser endomicroscopy, NDBE: non-dysplastic Barrett's esophagus, NDBO: non-dysplastic Barrett's oesophagus, LGD: low-grade dysplasia, HGD: high-grade dysplasia, BE: Barrett's esophagus

AI system category	Study	Data type	Data size
CADe system	Fockens et al. (2023) [6]	Neoplastic and NDBE images	1,713 neoplastic and 2,707 NDBE images from 1,229 patients
	de Groof et al. (2020a) [7]	Endoscopy images	Images from 10 patients with NDBE and 10 patients with Barrett's neoplasia
	de Groof et al. (2020b) [8]	Endoscopic images	494,364 labeled intestinal images, 1,704 unique esophageal images
	de Groof et al. (2019) [9]	WLE images	40 neoplastic and 20 NDBO images
	Abdelrahim et al. (2023) [10]	Neoplastic and non-neoplastic BE images/videos	75,198 images/videos (96 patients) and 1,014,973 images/videos (65 patients)
Deep learning on histology/biopsy	Faghani et al. (2022) [11]	Histology slides	NDBE, LGD, HGD slides
	Guleria et al. (2021) [12]	pCLE videos and biopsy images	1,970 pCLE videos, 897,931 biopsy patches, 387 whole-slide images
Real-time and video analysis	Hussein et al. (2022) [13]	Dysplastic and NDBE videos	148,936 video frames from 62 patients
	Hashimoto et al. (2020) [14]	Histology images	1,835 images from 64 patients
NLP and text-based systems	Nguyen Wenker et al. (2023) [15]	Pathology reports	600 for development, 400 for validation
Miscellaneous studies	Knabe et al. (2022) [16]	Images	1,020 images from 577 patients
	Ebigbo et al. (2021) [17]	Endoscopic images	230 images (108 T1a and 122 T1b)
	Swager et al. (2017) [18]	WLE images	60 WLE images from BE patients
	van der Sommen et al. (2016) [19]	Images	100 images from 44 patients

Computer-Aided Detection (CADe) Systems for Barrett's Esophagus

Empirical data from various studies examining the effectiveness of CADe systems in diagnosing Barrett's esophagus and related neoplasia are shown in Table 2. The sensitivity values reported across the studies are consistently high, ranging from 84% to 100%. In terms of specificity, the values vary between 64% and 90.7%, surpassing the capabilities of general endoscopists. These statistics underscore the diagnostic superiority of CADe systems when compared to general non-experienced endoscopists in these studies. Another study by de Groof et al. (2019) [9] introduces additional metrics, showcasing the CADe system's ability to localize neoplastic sites with 100% efficiency and assign a red flag score with 90% efficiency. The sample sizes of these studies also vary, ranging from as low as 20 to as high as 1,229, providing a broad scope for evaluating the performance of CADe systems.

**Table 2:** CADe systems for Barrett's esophagus and neoplasia CADe: computer-aided detection, NPV: negative predictive value

Study	Sample size	Sensitivity	Specificity	Other metrics	Conclusions
Fockens et al. (2023) [6]	1,229 patients	84%-100%	64%-66%	-	More sensitive than general endoscopists
Abdelrahim et al. (2023) [10]	161 patients	93%-95%	90%-94%	NPV: 95.1%, accuracy: 92%-94.7%	Outperforms non-expert endoscopists
de Groof et al. (2020a) [7]	20 patients	91%	89%	Accuracy: 90%	Detected Barrett's neoplasia with high accuracy
de Groof et al. (2020b) [8]	669 patients	93%	83%	Accuracy: 88%	Outperforms individual endoscopists
de Groof et al. (2019) [9]	60 patients	95%	85%	Localization and red flag score: 100% and 90%	High accuracy in detecting and localizing Barrett's neoplasia

Natural Language Processing (NLP) and Text-Based Systems

Another avenue gaining traction in the realm of Barrett's esophagus diagnosis is text-based systems, specifically NLP, as shown in Table 3. Nguyen Wenker et al. (2023) [15] focused on the development and validation of an NLP algorithm for identifying dysplasia in Barrett's esophagus patients through histopathology reports within a large integrated electronic medical record (EMR) system. The study by Nguyen Wenker et al. (2023) [15] featured a substantial sample size of 1,000 pathology reports and yielded remarkable results. In both the development and validation sets, NLP demonstrated outstanding performance, identifying dysplasia with an accuracy ranging from 98% to 98.7%. The sensitivity of the algorithm was also impressive, between 91.7% and 92.3%. Perhaps most noteworthy is the study's finding that NLP achieved 100% precision in the validation cohort.

**Table 3:** NLP and text-based systems for Barrett's esophagus NLP: natural language processing

Study	Sample size	Sensitivity	Other metrics	Conclusions
Nguyen Wenker et al. (2023) [15]	1,000	91.7%-92.3%	Accuracy: 98%-98.7%	High degree of sensitivity and accuracy

Application of Deep Learning on Histology and Biopsy Images for BE

The application of deep learning models to histology and biopsy images offers a transformative potential in the accurate and timely diagnosis of BE and its associated dysplasia. This is particularly significant given the interobserver disagreements often seen among pathologists, as well as the limitations of conventional diagnostic methods.

One notable study by Faghani et al. (2022) [11] focused on developing a deep learning model to specifically identify different grades of dysplasia in whole-slide images. Utilizing an ensemble approach, the study effectively combined the "You Only Look Once" (YOLO) model, achieving a specificity of 100%. The sensitivity for low-grade dysplasia (LGD) was 81.3%, while for non-dysplastic BE (NDBE) and high-grade dysplasia (HGD), it was over 90%. Notably, the study highlighted the F1 score (a measure that considers both sensitivity and specificity) ranging from 0.91 for NDBE to a perfect 1.0 for HGD.

Another comprehensive study by Guleria et al. (2021) [12] extended the scope of analysis to probe-based confocal laser endomicroscopy (pCLE) videos, biopsy patches, and whole-slide images. Remarkably, this study achieved an overall diagnostic accuracy of 90%, closely paralleling human diagnostic abilities. In terms of sensitivity, the deep learning models performed at 71% for pCLE videos and 72% for biopsy patches. The whole-slide-image-level model even achieved a high sensitivity of 90% for dysplasia. Table 4 summarizes these study findings as shown below.

**Table 4:** Deep learning models for histology and biopsy images in Barrett's esophagus diagnosis

Study	Sample size	Sensitivity	Specificity	Other metrics	Conclusions
Faghani et al. (2022) [11]	542	81.3%->90%	100%	F1 score: 0.91-1.0	Accurately identifies dysplasia grades
Guleria et al. (2021) [12]	Various types of diagnostic images	71%-90%	N/A	Overall accuracy: 90%	Mimics human-level diagnostic accuracy

Real-Time and Video Analysis for Barrett's Eesophagus

The advent of real-time and video analysis in the diagnosis and surveillance of BE marks a pivotal milestone in the enhancement of endoscopic procedures. These computational techniques offer unparalleled performance metrics, revolutionizing the way clinicians detect and manage dysplastic lesions and early neoplasia.

For example, a groundbreaking study by Hussein et al. (2022) [13] leveraged convolutional neural networks (CNNs) to identify areas of dysplasia, thereby informing targeted biopsy procedures. This CNN model demonstrated compelling results: an area under the curve (AUC) of 93% with an impressively fast assessment speed of 48 frames per second (fps). Furthermore, the model achieved a sensitivity of 91% and a specificity of 79% in dysplasia detection.

Another innovative study by Hashimoto et al. (2020) [14] employed CNN-based object detection algorithms to provide high-accuracy, real-time detection of early esophageal neoplasia. The model achieved a sensitivity of 96.4%, a specificity of 94.2%, and a mean average precision of 0.7533, reinforcing the algorithm's capability for early, precise detection. Table 5 summarizes these study findings as shown below.

**Table 5:** Real-time and video analysis for Barrett's esophagus AUC: area under the curve, fps: frame per second, BE: Barrett's esophagus

Study	Sample size	Sensitivity	Specificity	Other metrics	Conclusions
Hussein et al. (2022) [13]	79	91%	79%	AUC: 93%, speed: 48 fps	Excellently supports endoscopic surveillance of BE
Hashimoto et al. (2020) [14]	65	96.40%	94.20%	Mean average precision: 0.7533	High efficacy in real-time detection of early neoplasia

Miscellaneous Studies

A pivotal study by Knabe et al. (2022) [16] demonstrated an impressive overall accuracy rate of 73% in the classification of T stages in Barrett's carcinoma. This study holds particular clinical significance as the T stage serves as a major determinant for subsequent treatment approaches. Notably, this study employed a CNN trained and internally validated on 1,020 endoscopic images from 577 patients with Barrett's adenocarcinoma. Such high levels of accuracy suggest that AI systems have significant potential to support endoscopists in making informed clinical decisions.

Similarly, another investigation by Ebigbo et al. (2021) [17] showcased that machine learning algorithms could achieve diagnostic performance comparable to that of international experts in the field. Specifically, this study focused on the challenging task of differentiating between T1a and T1b stages in Barrett's esophagus-related cancer. Utilizing deep learning algorithms, the study analyzed 230 white light endoscopic images and found no statistically significant difference in performance metrics such as sensitivity, specificity, and overall accuracy between the AI system and human experts. These findings are particularly encouraging as they reinforce the reliability and clinical applicability of AI-assisted diagnostic systems, summarized in Table 6.

**Table 6:** Miscellaneous studies AUC: area under the curve

Study	Sample size	Sensitivity	Specificity	Other metrics	Conclusions
Knabe et al. (2022) [16]	577	72%	64%	Overall accuracy: 73%	High accuracy in identifying T stage
Ebigbo et al. (2021) [17]	230	77%	64%	F1: 0.74, accuracy: 71%	Scored equally to international experts
Swager et al. (2017) [18]	60	90%	93%	AUC: .95	Good performance, needs in vivo validation
van der Sommen et al. (2016) [19]	44	86%	87%	N/A	Feasible, more research needed for real-time operation

Discussion

The advancement of artificial intelligence (AI) in diagnosing and managing Barrett's esophagus (BE) demonstrates considerable potential. Multiple methodologies, including computer-aided detection (CADe) systems, natural language processing (NLP), deep learning algorithms, and real-time and video Analysis, have shown impressive strides in enhancing diagnostic accuracy and efficiency [6,11,13,15]. This review aims to critically evaluate the empirical evidence supporting the use of these AI-based diagnostic systems in BE.

A key highlight from this review is the consistently high sensitivity rates exhibited by AI-based diagnostic systems, often exceeding 80% and reaching up to 100% in some cases [7,9,10]. Traditional endoscopic methods have often lagged in sensitivity, thereby supporting the integration of AI algorithms for improving early detection rates [6]. Although specificity also showed promising results, there is room for further improvement in this area [10].

The ability of AI systems to offer advanced diagnostic markers is particularly noteworthy. For instance, CAD systems show capabilities in "localization and red flag score," providing a more nuanced understanding of the pathology [9]. These added dimensions could be critical in shaping subsequent treatment plans and improving patient care.

Text-based NLP systems have exhibited extraordinary accuracy and sensitivity in identifying dysplasia from histopathology reports within electronic medical record (EMR) systems [15]. The use of NLP could significantly streamline the diagnostic process, reducing the time and potential errors associated with the manual review of medical records.

The application of deep learning models in BE diagnostics addresses the limitations of traditional methods and tackles interobserver disagreements commonly seen among pathologists [11,12]. The capability for real-time and video analysis furthers the sophistication of diagnostic tools, offering rapid assessments that can aid in immediate clinical decision-making during endoscopic procedures [13,14].

The potential for AI-based methods to assist in staging, as seen in the study represented by Knabe et al. (2022) [16], adds another layer of clinical utility. Equally significant is the finding that machine learning algorithms can match the performance of international experts in the field, reducing the scope for human error in diagnosis [17].

Despite the promising advancements, several limitations warrant attention. The effectiveness of AI systems in multicenter trials and more diverse populations has yet to be fully substantiated [7]. Moreover, issues around data privacy, ethical implications, and the requirement for specialized computational infrastructure remain challenges for broader implementation.

## Conclusions

AI-based methods represent a revolutionary shift in the diagnostic landscape for Barrett's esophagus. These technologies offer superior sensitivity and specificity while also enhancing clinical decision-making through comprehensive diagnostic metrics. Additional research is needed to address existing limitations and facilitate the translation of these advancements from bench to bedside.
